# Supplementary material for: Learning enhances encoding of time and temporal surprise in mouse primary sensory cortex
Source: Nat Commun. 2022 Sep 20;13:5504. doi: 10.1038/s41467-022-33141-y (PMC9489862; doi:10.1038/s41467-022-33141-y)
Supplement: Supplementary file 1 — Supplementary Information [file 41467_2022_33141_MOESM1_ESM.pdf]

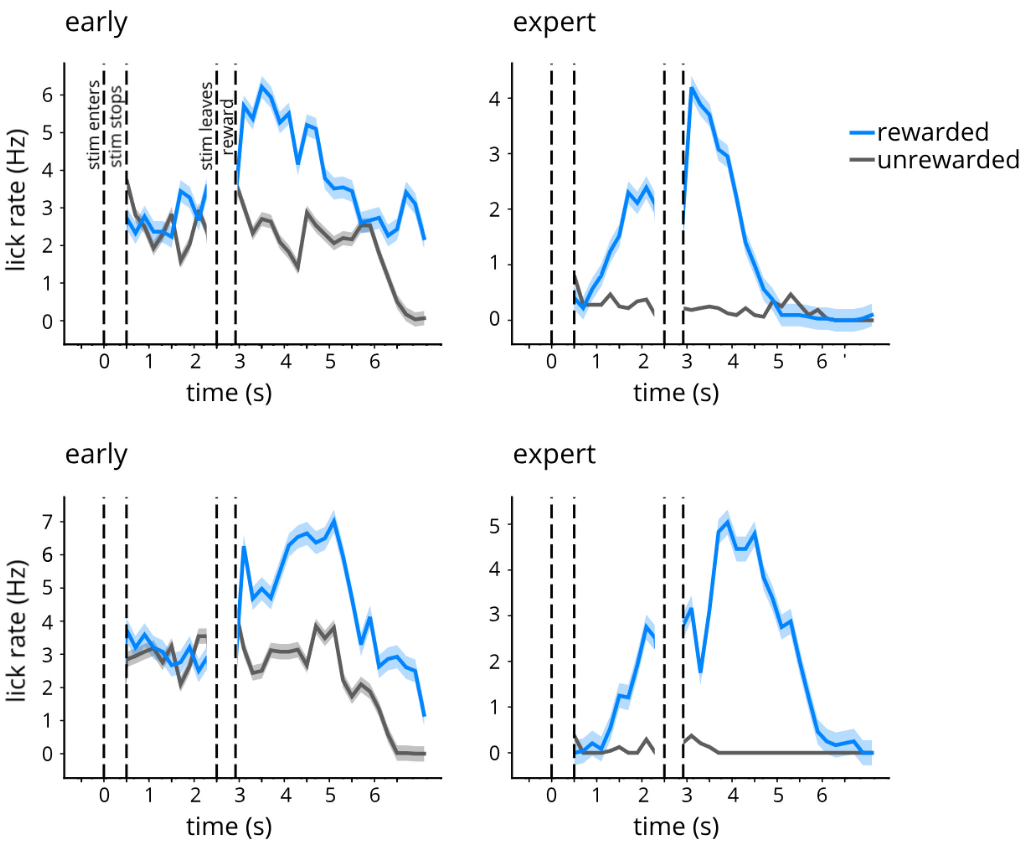

Supplementary Figure 1: Licking behavior of two example mice.
Each row corresponds to an example mouse. Left: early sessions; right: expert sessions. Rewarded trials are shown in blue; unrewarded trials in grey. Shading corresponds to SEM.

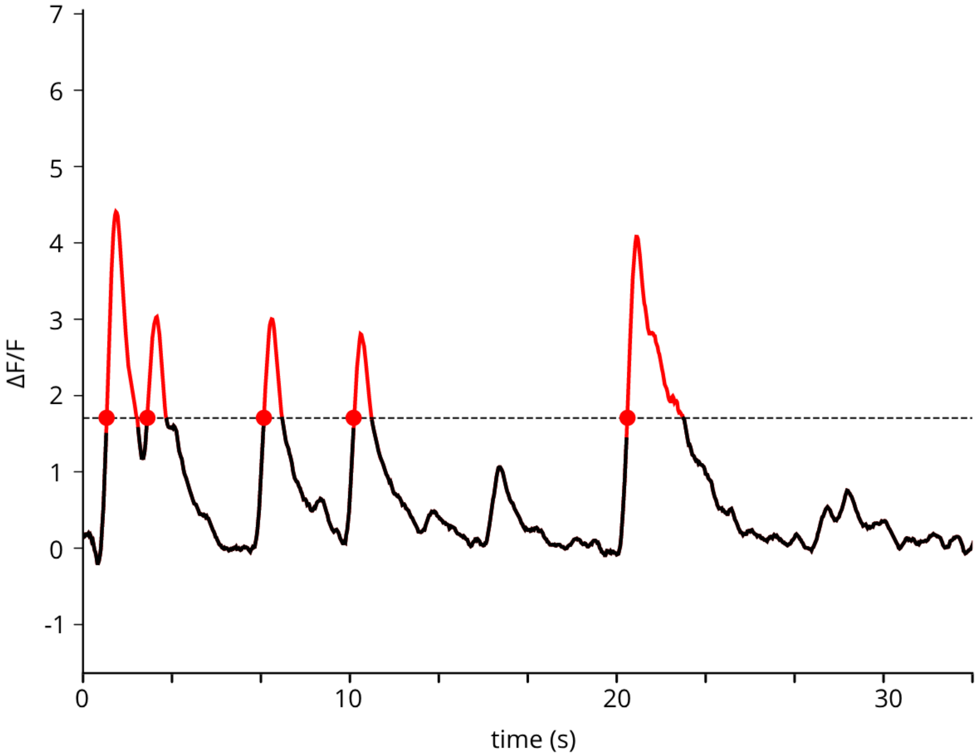

Supplementary Figure 2: Detecting calcium transients from fluorescence.
Threshold for each cell is set as 2 standard deviations above the median baseline-corrected fluorescence (ΔF/F) for that cell. The time when the trace first crosses the threshold is considered to be the transient onset time. Suprathreshold periods are colored red; transient onsets are marked with red circles.

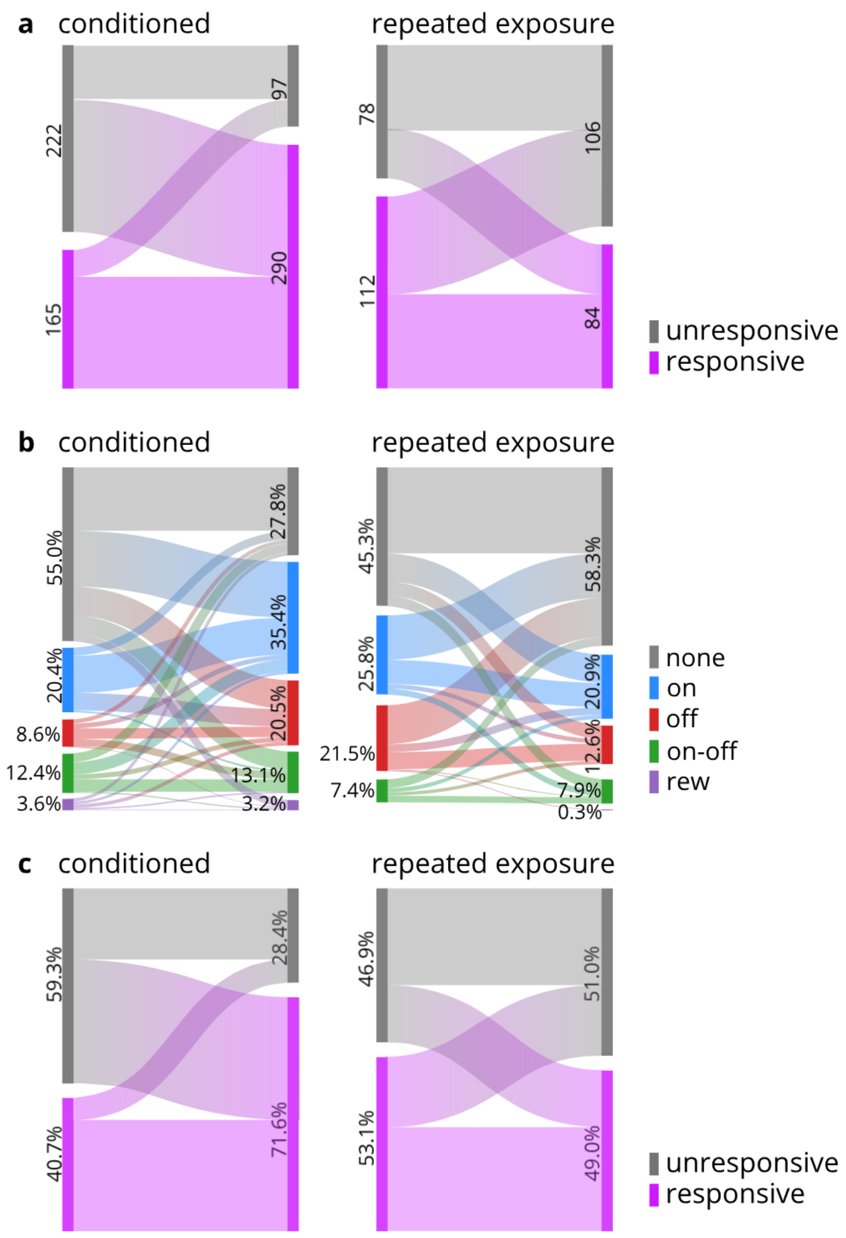

Supplementary Figure 3: Longitudinally tracked cells become more responsive after conditioning, but not repeated exposure.

a) Same as main Fig 3c, except cells from all mice are pooled together (rather than averaged across mice).

b) Same as Fig 3c, but splitting responsive cells into response sub-types (on, off, on-off, and reward). Note that there is no reward in the repeated exposure condition, but on the late day (right plot), 0.3% of cells exhibit a “late” response, so they are indistinguishable from the “reward” group.

c) Same as Fig 3c, but excluding any ROIs that on any given the expert day overlap with more than one ROI from the early day (and the reverse).

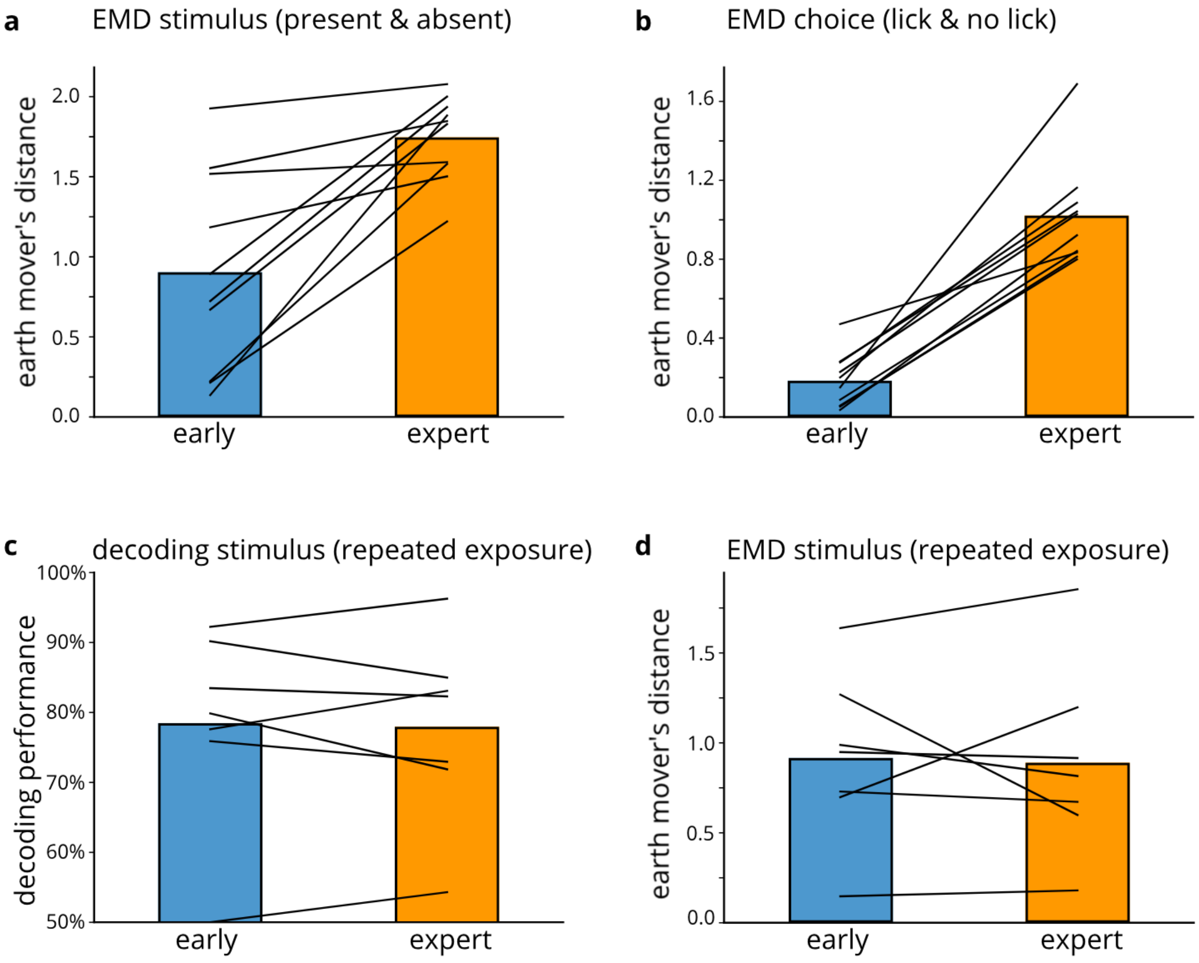

Supplementary Figure 4: neural representations of trial types diverge with learning, but not following repeated exposure.

a) Earth mover's distance (EMD) between stimulus classes (present & absent) on early and expert days. Black lines indicate individual mice; bars are the average. $p = 0.005$ (two-sided Wilcoxon signed rank test). For this and remaining panels, data is provided in Source Data file.

b) Same as (a) but for choice classes (lick & no lick). $p = 0.005$.

c) Decoding stimulus from cell population of repeated exposure mice on early (blue) and late (orange) days. $p = 0.925$ (two-sided paired t-test).

d) Same as (a) but for repeated exposure mice.

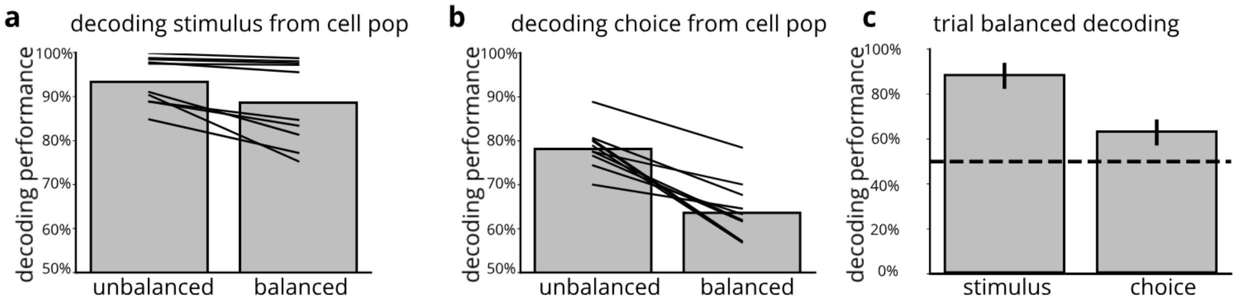

Supplementary Figure 5: Trial-balanced decoding performance is lower than unbalanced approach, but still above chance.

a) Trial-balanced stimulus decoding performance (right) compared to unbalanced (left) ($p=0.03$). Source data is provided in Source Data file.

b) Same as (a) but for choice ($p = 0.0002$).

c) Mean decoding performance for trial balanced decoding, with 95% confidence interval indicated with the vertical line ($n = 10$ mice). Note that 95% confidence interval for trial-balanced decoding (for stimulus and choice) is above chance.

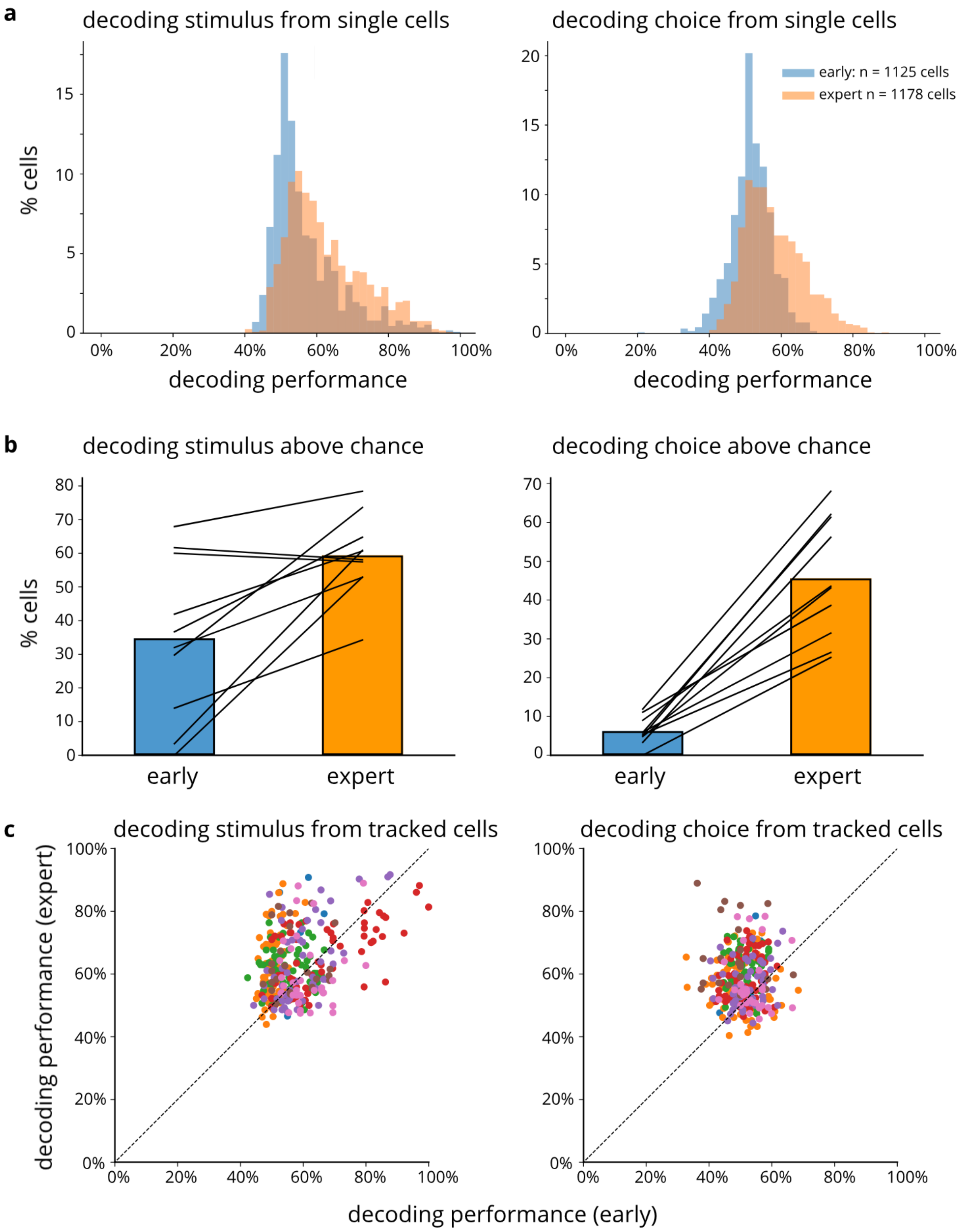

Supplementary Figure 6: Training enhances decodability of stimulus and choice, from activity of individual cells.

a) Histograms of the percent of cells from which can decode stimulus (left) and choice (right) at a given accuracy, on naïve (blue) and expert (orange) days.

b) Percent of cells from which can stimulus (left) and choice (right) can be decoded above chance, on naïve (blue) and expert (orange) days (stimulus $p = 0.004$; choice $p = 2.33 \times 10^{-5}$, two-sided paired t-test). Data is provided in Source Data file.

c) Decoding performance for stimulus (left) and choice (right), from individual longitudinally tracked cells, on naive vs expert days (stimulus $p = 2.17 \times 10^{-12}$; choice $p = 5.89 \times 10^{-28}$, two-sided paired t-test). Colors correspond to cells from different mice.

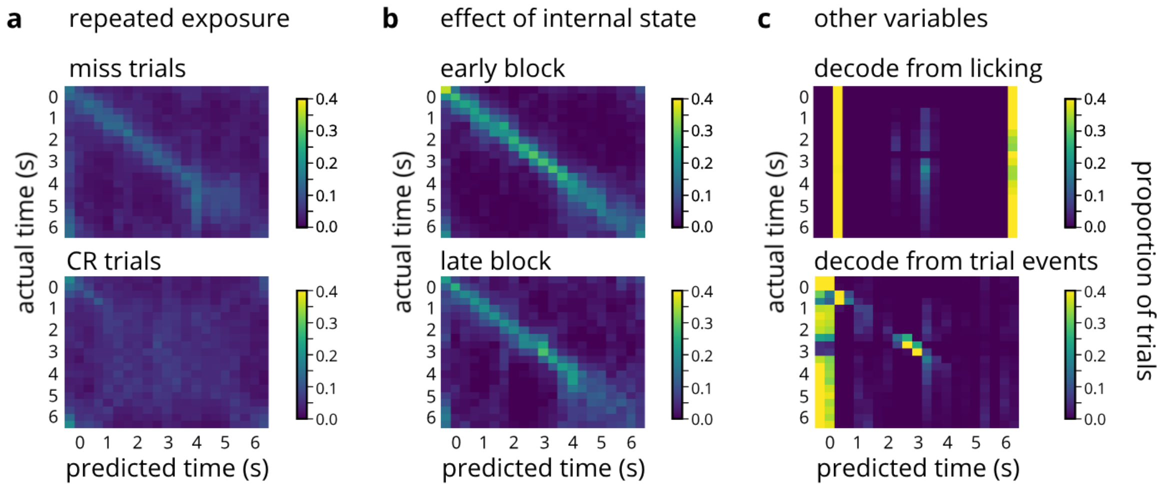

Supplementary Figure 7: Characteristics of time decoding.
a) Poor time encoding in repeated exposure mice (n = 7 mice).
b) Internal state (thirsty vs. satiated) has no effect on tome coding. Top: time decoding from first third of trials in each session. Bottom: time decoding from last third of trials in each session (n = 10 mice).
c) Time progression cannot be decoded from licking (top) or trial events (bottom). Trial events include stimulus onset, stimulus offset, reward, licking, and whisking (n = 10 mice).

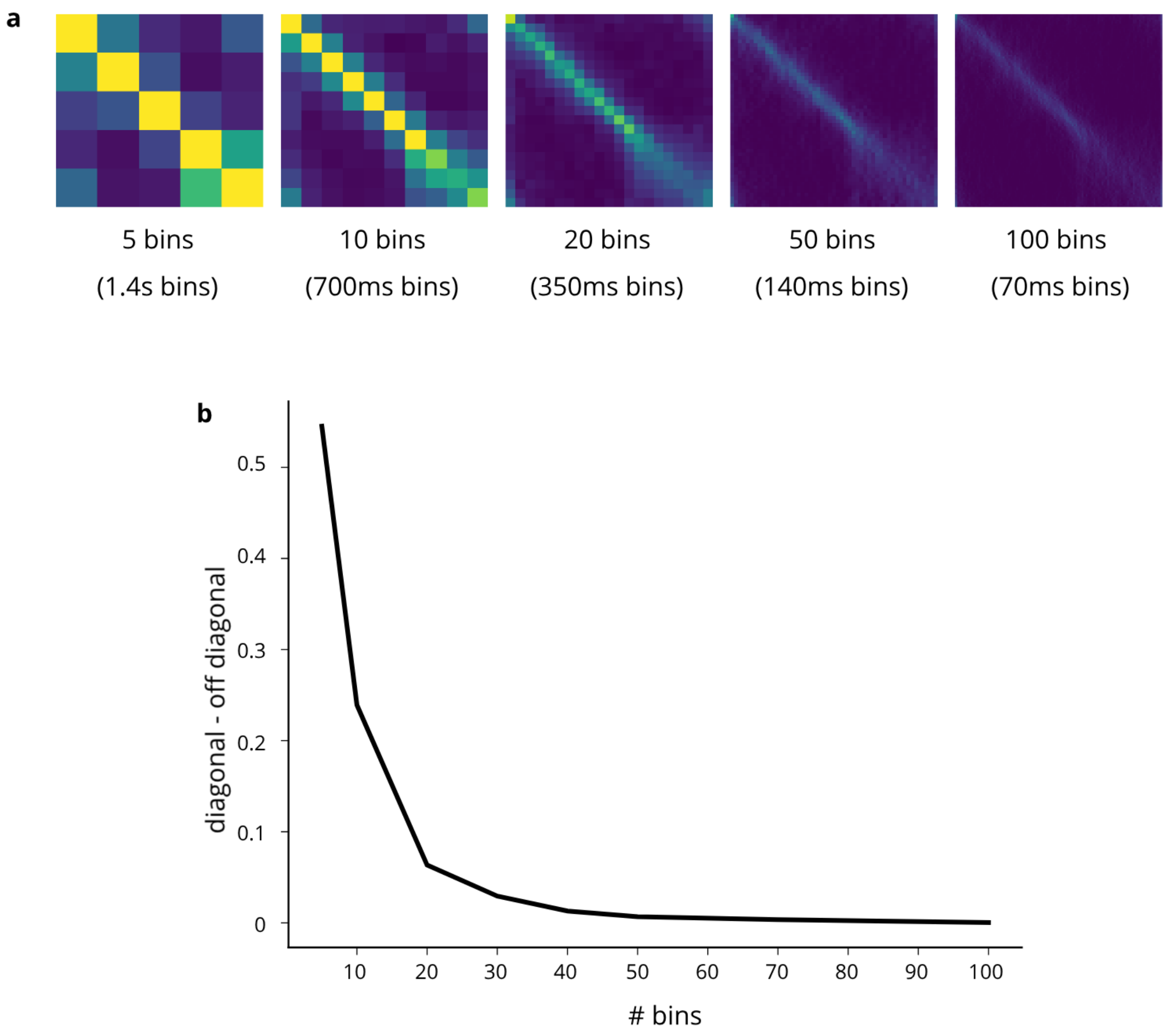

Supplementary Figure 8: Varying time bin size reveals resolution of encoded temporal information.
a) Confusion matrices illustrating time decoding for different bin sizes.
b) Subtracting the confusion matrix off-diagonal from the diagonal provides a measure of the resolution of encoded temporal information. The y-axis is the average difference between the value of a time bin on the diagonal and values of the adjacent time bins (mean of left and right off-diagonal time bins); therefore, the units are a difference between proportions (since the value of a given time bin is the proportion of trials on which time y is predicted to be time x). The curve asymptotes near y=0, when neighboring time bins can no longer be distinguished.

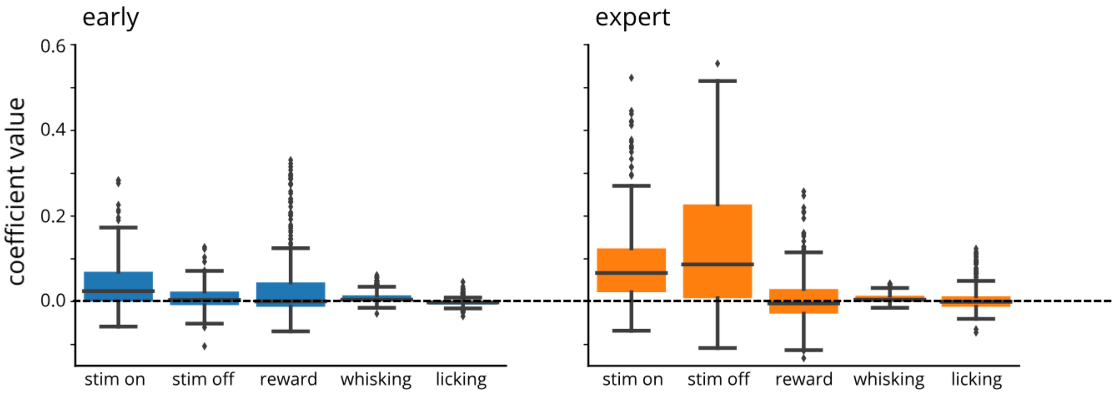

Supplementary Figure 9: Contribution of various possible factors to neuronal activity.
Plotted are coefficients of various features that were part of linear regression models of neuronal activity on early (left) and expert (right) sessions. Features consisted of stimulus onset, stimulus offset, reward, whisking motion, and licking. Note that movements (whisking and licking) have near-zero contributions to the regression model even on the expert day. Each box-plot shows the median coefficient value for the feature on the x-axis; the ends of the colored box show the first and third quartile; the “whiskers” extend down to the minimum and up to the maximum, with dots indicating outliers. Early session: n = 234 cells across 3 mice. Expert session: n = 209 cells across 3 mice. Data is provided in Source Data file.

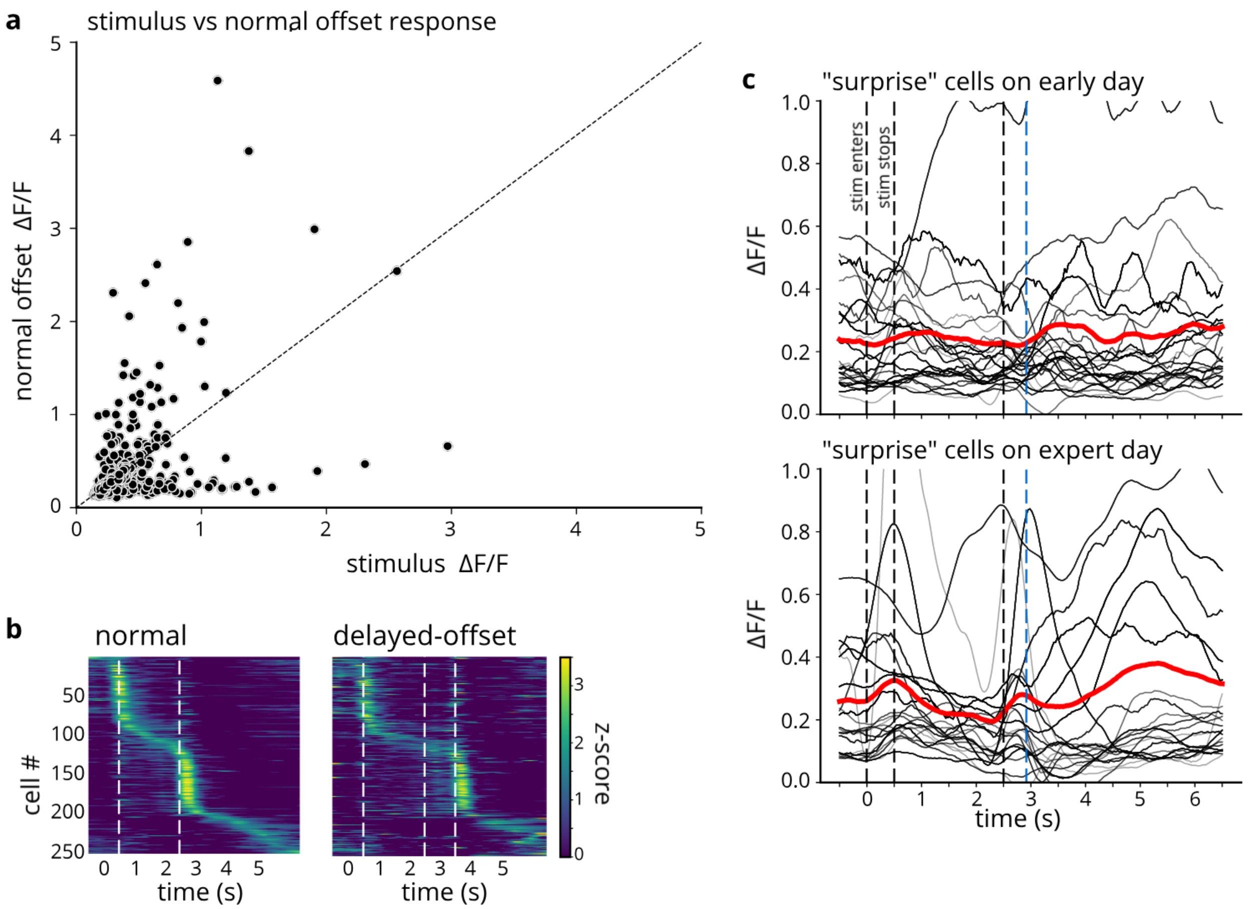

Supplementary Figure 10: Characterization of cells that respond to delayed offset.

a) Response amplitude to stimulus onset vs the response to stimulus offset for normal trials ($p = 0.001$). Data is provided in Source Data file.

b) Left: heatmap of all cells (3 mice) sorted by maximum response on normal trials. Right: same cells, in the same order, but with responses on the delayed offset trials shown.

c) Longitudinally tracked “surprise” cells on an early training day (top) and on an expert day (bottom). Red trace is the mean across cells.

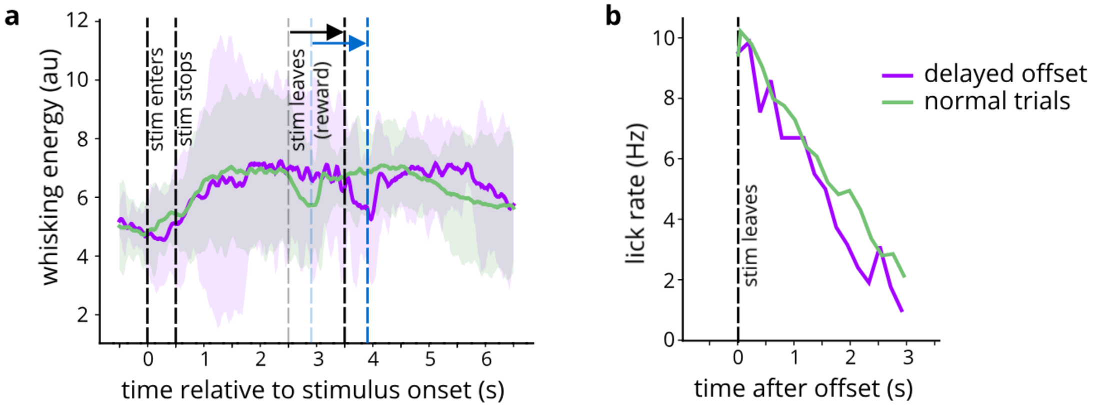

Supplementary Figure 11: Comparable whisking and licking on normal vs delayed-offset trials.

a) Whisking patterns on normal (green) and delayed-offset (purple) trials. Solid lines are the mean values, and shaded area corresponds to 95% confidence interval.

b) Licking behavior on normal (green) and delayed-offset (purple) trials, aligned to the time of stimulus offset.
