# Supplementary material for: Learning enhances encoding of time and temporal surprise in mouse primary sensory cortex
Source: Nat Commun. 2022 Sep 20;13:5504. doi: 10.1038/s41467-022-33141-y (PMC9489862; doi:10.1038/s41467-022-33141-y)
Supplement: Supplementary file 3 — Reporting Summary [file 41467_2022_33141_MOESM3_ESM.pdf]

nature portfolio

Corresponding author(s): Randy Bruno

Last updated by author(s): 08/09/2022

Reporting Summary

Nature Portfolio wishes to improve the reproducibility of the work that we publish. This form provides structure for consistency and transparency in reporting. For further information on Nature Portfolio policies, see our Editorial Policies and the Editorial Policy Checklist.

Statistics

For all statistical analyses, confirm that the following items are present in the figure legend, table legend, main text, or Methods section.

n/a	Confirmed	
☐	☒	The exact sample size (n) for each experimental group/condition, given as a discrete number and unit of measurement
☐	☒	A statement on whether measurements were taken from distinct samples or whether the same sample was measured repeatedly
☐	☒	The statistical test(s) used AND whether they are one- or two-sided<br>*Only common tests should be described solely by name; describe more complex techniques in the Methods section.*
☒	☐	A description of all covariates tested
☐	☒	A description of any assumptions or corrections, such as tests of normality and adjustment for multiple comparisons
☐	☒	A full description of the statistical parameters including central tendency (e.g. means) or other basic estimates (e.g. regression coefficient) AND variation (e.g. standard deviation) or associated estimates of uncertainty (e.g. confidence intervals)
☐	☒	For null hypothesis testing, the test statistic (e.g. F, t, r) with confidence intervals, effect sizes, degrees of freedom and P value noted<br>*Give P values as exact values whenever suitable.*
☒	☐	For Bayesian analysis, information on the choice of priors and Markov chain Monte Carlo settings
☒	☐	For hierarchical and complex designs, identification of the appropriate level for tests and full reporting of outcomes
☒	☐	Estimates of effect sizes (e.g. Cohen's d, Pearson's r), indicating how they were calculated

Our web collection on statistics for biologists contains articles on many of the points above.

Software and code

Policy information about availability of computer code

Data collection	ScanImage (5.7)
Data analysis	Suite2p (0.5.5); python 2.7; scikit-learn; OpenCV

For manuscripts utilizing custom algorithms or software that are central to the research but not yet described in published literature, software must be made available to editors and reviewers. We strongly encourage code deposition in a community repository (e.g. GitHub). See the Nature Portfolio guidelines for submitting code & software for further information.

Data

Policy information about availability of data

All manuscripts must include a data availability statement. This statement should provide the following information, where applicable:

- Accession codes, unique identifiers, or web links for publicly available datasets
- A description of any restrictions on data availability
- For clinical datasets or third party data, please ensure that the statement adheres to our policy

Due to the size of imaging data, data will be available upon request from the authors

Field-specific reporting

Life sciences study design

All studies must disclose on these points even when the disclosure is negative.

Sample size	Sample sizes were chosen not by statistical methods but rather to be comparable to sample sizes of other studies in the field (see citations in Methods section).
Data exclusions	No data were excluded.
Replication	Multiple mice are included in the dataset specifically to address this issue. 10 mice were used in the conditioned cohort, and 7 were used in the repeated exposure group.
Randomization	Pure randomization was used in selecting trial type. Both trials and experimental groups were randomized: the trial type was selected randomly for each trial, and animals were allocated to the conditioned and repeated exposure groups at random.
Blinding	Blinding is not possible for acquiring data from repeated exposure versus conditioning groups. It is obvious to both the experimenter and the mouse whether or not the animal has been assigned to one group or the other: repeated exposure mice did not receive water rewards during the task, so the experimenter had to ensure that this cohort of mice received water outside of the task context; in addition, water restricted mice weighed less.

Reporting for specific materials, systems and methods

We require information from authors about some types of materials, experimental systems and methods used in many studies. Here, indicate whether each material, system or method listed is relevant to your study. If you are not sure if a list item applies to your research, read the appropriate section before selecting a response.

Materials & experimental systems

n/a	Involved in the study	
☒	☐	Antibodies
☒	☐	Eukaryotic cell lines
☒	☐	Palaeontology and archaeology
☐	☒	Animals and other organisms
☒	☐	Human research participants
☒	☐	Clinical data
☒	☐	Dual use research of concern

Methods

n/a	Involved in the study	
☒	☐	ChIP-seq
☒	☐	Flow cytometry
☒	☐	MRI-based neuroimaging

Animals and other organisms

Policy information about studies involving animals; ARRIVE guidelines recommended for reporting animal research

Laboratory animals	C57BL/6J mice; male and female; age range p60-p300
Wild animals	no wild animals used
Field-collected samples	no samples were collected from the field
Ethics oversight	Columbia University Institutional Animal Care and Use Committee

Note that full information on the approval of the study protocol must also be provided in the manuscript.
